# Active transport enables protein condensation in cells

**DOI:** 10.1126/sciadv.adv7875

**Published:** 2025-05-23

**Authors:** Gaurav Chauhan, Edward G. Wilkinson, Yaning Yuan, Samuel R. Cohen, Masayuki Onishi, Rohit V. Pappu, Lucia C. Strader

**Affiliations:** ^1^ Department of Biomedical Engineering and Center for Biomolecular Condensates, James McKelvey School of Engineering, Washington University in St. Louis, St. Louis, MO 63130, USA.; ^2^ Department of Biology, Duke University, Durham, NC 27708, USA.

## Abstract

Multiple factors drive biomolecular condensate formation. In plants, condensation of the transcription factors AUXIN RESPONSE FACTOR 7 (ARF7) and ARF19 attenuates response to the plant hormone auxin. Here, we report that actin-mediated movement of cytoplasmic ARF condensates enhances condensation. Coarse-grained molecular simulations of active polymers reveal that applied forces drive the associations of macromolecules to enhance phase separation while giving rise to dense phases that preferentially accumulate motile molecules. Our study highlights how molecular motility can drive phase separation, with implications for motile condensates while offering insights into cellular mechanisms that can regulate condensate dynamics.

## Teaser

The force generated by active transport modulates protein condensation.

## INTRODUCTION

Biomolecular condensates play pivotal roles in the spatial and temporal control of cellular processes (*1*) to affect nearly every cell function. Formation and dissolution of condensates are highly sensitive to changes in temperature, pH, and salt (*2*). The reversible nature of condensate formation allows for dynamic regulation of cellular activities, yet it is unclear whether we have unmasked all possible drivers of condensation.

Plant growth and development are controlled by pathways that respond to the phytohormone auxin (*3*, *4*). Nucleocytoplasmic partitioning of AUXIN RESPONSE FACTORs (ARFs) such as ARF7 and ARF19 controls auxin transcriptional responses in *Arabidopsis thaliana* (*5*, *6*)*.* ARF7 and ARF19 are recently diverged transcription factors with redundant functions in auxin signaling (*7*). These two proteins are thought to have nearly identical roles in plant development and are hence interoperable with one another.

Attenuation of transcriptional activity is achieved by sequestering ARF7 and ARF19 in cytoplasmic condensates via a process referred to as condensation (*8*) that combines binding, polymerization, percolation, and phase separation (*5*). The formation of cytoplasmic condensates of ARFs is driven in part by the polymerization of the PB1 (Phox and Bem1) domain (*9*, *10*). Polymerization generates emergent multivalence of intrinsically disordered prion-like low complexity domains (*11*) such that above a threshold concentration, referred to as the saturation concentration (*c*_sat_), the ARFs undergo phase separation, giving rise to condensates.

## RESULTS

**Fig. 1.**
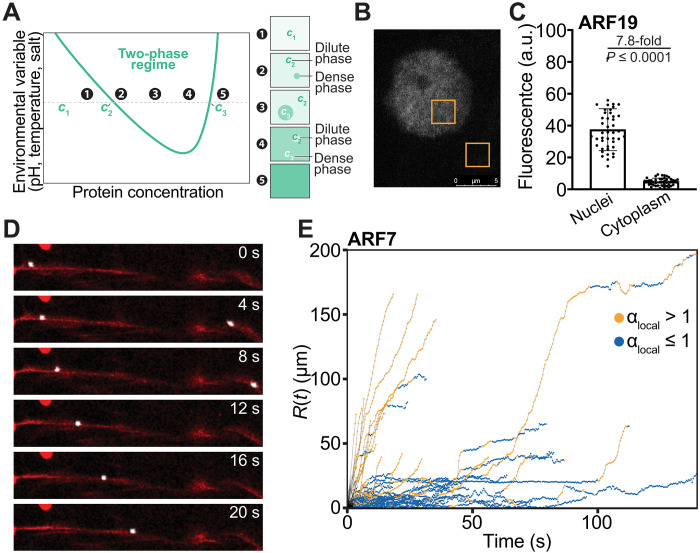
ARF condensates form in the cytoplasm and undergo active motion. (**A**) Schematic of the phase diagram for systems undergoing thermoresponsive phase separation. The green curve represents the phase boundary. For a given temperature, concentrations 2, 3, and 4 lie within the two-phase regime. (**B**) Representative image showing the fluorescence intensity of ARF19 in the nucleus versus the cytoplasm. (**C**) Quantification of the dilute phase fluorescence intensities for ARF19 in the nucleus versus the cytoplasm. a.u., arbitrary units. (**D**) Time-lapse images of an ARF condensate (white) moving along the LifeAct-tagged actin filaments (red) in the cytoplasm during transient expression in tobacco. (**E**) Analysis of local MSDs over short-time intervals following the approach of Arcizet *et al.* (*17*) Here, the trajectories *R*(*t*) is defined as *R*(*t*) = 〈[*r*(*t*) − *r*_0_]^2^〉^0.5^, where *r*(*t*) is the position of the condensate at time *t* and *r*_0_ is the position at *t* = 0. The trajectories, shown here for ARF7 condensates, are colored on the basis of the exponent for the MSDs evaluated over short-time windows.

In contrast to the cytoplasm, ARF condensates do not persist within the interior of the nucleus (*5*, *12*). If ARF condensation is controlled purely by passive processes, then the implication would be that the concentrations of ARFs in the nucleus must be below their intrinsic *c*_sat_ (Fig. 1A). To test this hypothesis, we quantified the concentrations of ARF19 in the nucleoplasm and the dilute phase of the cytoplasm (Fig. 1B), finding that the ARF19 concentration is higher in the nucleoplasm than in the dilute phase of the cytoplasm (Fig. 1C). Despite this, ARF19 condensates are not often observed in the nucleus (movie S1). This observation is suggestive of one of four implications: (i) There likely are active drivers of condensation in the cytoplasm; (ii) condensation within the nucleus is likely inhibited by active processes; (iii) nuclear as opposed to cytoplasmic ligands bind preferentially to ARFs in the dilute phase to weaken or suppress phase separation via the mechanism of polyphasic linkage (*13*, *14*); (iv) or the solution conditions in the nucleoplasm and the cytosol are very different from one another, implying that the driving forces for phase separation via purely passive considerations, encoded by two- and three-body interaction coefficients (*15*, *16*), are very different in the two milieus. We pursued option (i) because clues to the presence of active drivers of phase separation in the cytoplasm were evident in the observation that ARF condensates move along actin filaments (Fig. 1D and movie S2). To test whether the motions of condensates are concordant with active transport, we analyzed the motions of condensates by quantifying how the local mean squared displacement (MSD) scales with time (*t*) (*17*). Purely passive motion should be diffusive, implying that MSD ∝ *t*^α^, with α = 1 (*17*). We observed a combination of trajectories, with a substantial fraction being defined by values of α greater than unity. This implies that the motions of ARF condensates in the cytoplasm are superdiffusive (Fig. 1E) and likely to be driven by motors.

**Fig. 2.**
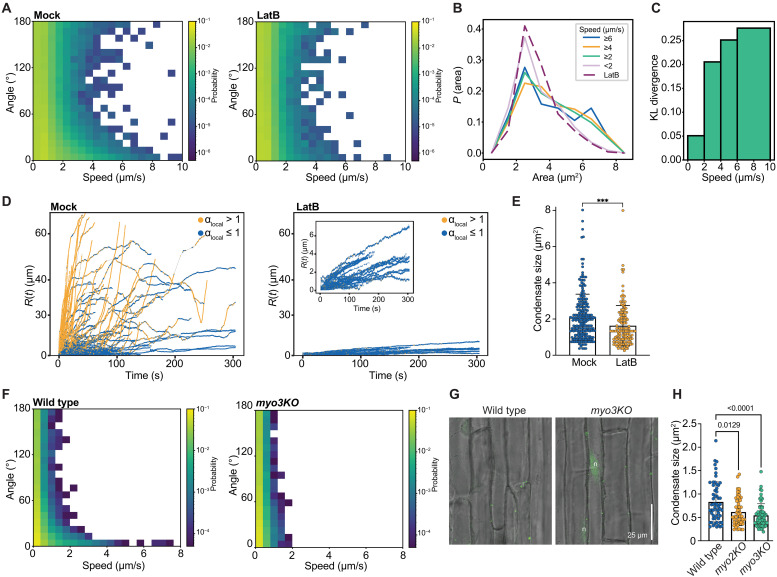
Condensate motility is aided by the actin network. (**A**) Joint probability densities for angles and speeds of the tracked pARF19:ARF19-mVenus condensates from seedlings incubated for 2 hours in a mock (DMSO) or 10 μM LatB treatment. Data collected using TrackMate (*58*) are shown for five distinct condensates captured from immature trichoblasts over the course of 5 min at a frame rate of 2.3 frames/s. (**B**) Marginal probability densities for areas of condensates were computed for condensates corresponding to different speed intervals. These were compared to the distributions obtained upon LatB treatment (dashed curve). For high-speed condensates, we observe a pronounced shift toward condensates of large areas. (**C**) KL divergence computed for each distribution as the posterior and the distribution obtained from LatB treatment as the prior. (**D**) Distance *R*(*t*) traversed by ARF19 condensates at time *t* after a 2-hour treatment with mock (DMSO) or 10 μM LatB. Local scaling of MSD with lag time is used to color the trajectories based on the exponents. Values of α > 1 indicate active motion. (**E**) Area of condensates after a 10-min treatment with mock (DMSO) or LatB. (**F**) Angle and speed distributions for YFP-ARF19 condensates in young root epidermal cells of wild-type and *myo3KO* seedlings carrying *pUBQ10:YFP-ARF19*. (**G**) Confocal images of young root epidermal cells of wild-type and *myo3KO* seedlings carrying *pUBQ10:YFP-ARF19*. (**H**) Mean YFP-ARF19 condensate areas (±SD) in immature trichoblasts of wild type (Col-0), *myo2KO*, or *myo3KO* carrying *pUBQ10:YFP-ARF19*.

Cytoplasmic ARF19 condensates move at a range of speeds, with the highest speeds of ~10 μm/s (Fig. 2A). These speeds are coincident with low angles that collectively suggest ballistic and directional motions (Fig. 2A). The high speeds we observed suggest that movement could be driven by myosin XI motors. Assuming that the condensate speeds serve as proxies for the speeds of the myosin motors, the speeds we observe make them among the fastest known myosins (*18*). This combination of high speeds and low angles is a signature of active transport (*17*). The high-speed directional motion of ARF19 condensates is facilitated by the actin cytoskeleton; latrunculin B (LatB) treatment, which depolymerizes actin filaments (*19*, *20*), diminished ARF19 condensate movement (Fig. 2A and movie S2). However, unlike LatB treatment, disruption of microtubules by oryzalin (*21*) does not disrupt the movement of ARF condensates (fig. S1). From the joint histograms, we computed a set of histograms of condensate sizes. Each histogram corresponds to condensates that move with specific speeds. The data (Fig. 2B) show that the condensates observed upon LatB treatment are smaller in size when compared to the condensates observed in untreated cells. We computed a Kullback-Leibler (KL) divergence to quantify the divergence between different area histograms, each quantified for condensates of different speeds, and the area histogram observed under LatB treatment (Fig. 2C). This quantity computes the divergence between distributions in terms of a cross entropy. We use the distributions for the untreated cells as the prior and those extracted from treated cells as the posterior. The KL divergence essentially computes a non-Euclidean distance between the prior and the posterior. A low value for the KL divergence implies high similarity between the posterior and the prior, whereas larger KL divergence values imply lower similarities. We observed large KL divergences for high-speed condensates as opposed to low-speed ones, implying that condensates form via a combination of passive and active forces. The latter enhances condensation as evidenced by the preference for larger condensates when the speeds of the condensates increase.

We then studied the displacements of condensates as a function of time by analyzing the trajectories followed by ARF19 condensates (Fig. 2D). In untreated cells, trajectories defined by α > 1 coexist with trajectories defined by α ≤ 1. In contrast, LatB treatment leads to a dominance of trajectories defined by exponents with values of α ≤ 1. As noted above, the KL divergence is indicative of the condensate pool comprising a combination of active and passive species. We put this on a quantitative footing using the information in Fig. 2D to extract the number of occurrences of ARF19 condensate movements showing α < 1 and α > 1. We observed ~32% active condensates with an exponent of α > 1 for the local MSD, showing active movement. For the pARF7 data in Fig. 1E, we observed ~29% active condensates with an exponent of α > 1 for the local MSD.

Condensate size provides a useful proxy for the driving forces for condensation (*22*). If the actin cytoskeleton serves as an elastic network that inhibits condensate ripening without influencing the driving forces for condensation (*23*, *24*), then LatB treatment should lead to larger condensates. Conversely, if the driving forces for condensation are enhanced by movements along the actin cytoskeleton, then LatB treatment should lead to smaller condensate sizes. We find that short-term LatB treatment leads to condensates that are smaller in size when compared to the mock treatment (Fig. 2E and fig. S3A), implying that disruption of the actin cytoskeleton weakens the driving forces for condensation.

Actin-dependent movement of ARF19 condensates hints at a role for myosin-dependent trafficking of ARF19. We found that ARF19 condensate motility was ablated in the *myosin xi-k xi-1 xi-2* (*myo3KO*) mutant background (Fig. 2F, figs. S3B and S4, and movie S3). This result implicates a role for the three myosin motor proteins in the ARF19 condensate movement. Consistent with our results with LatB (Fig. 2E), we found that ARF19 condensates were smaller in the *myo3KO* background than in wild type (Fig. 2, G and H).

Together, our data demonstrate that ARF condensates are actively transported in the cytoplasm, powered by myosin motors along the actin cytoskeleton. This myosin-aided movement influences the sizes of condensates (Fig. 2, E and H). These observations raise the possibility that motility augments the passive contributions to condensation. To understand and model how applied force influences motility and condensation, we turned to Langevin dynamics simulations of polymers that can undergo spontaneous phase separation (*25*).

### Simulations show how motility enables phase separation

**Fig. 3.**
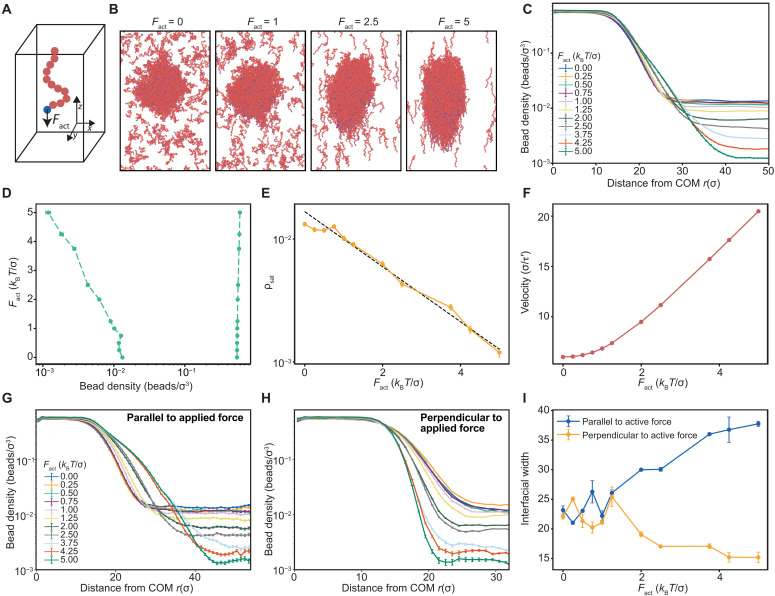
Simulations show how motility enhances the driving forces for condensation. (**A**) Langevin dynamics simulations were deployed to study the effects of forces applied along the *z*-direction to each polymer in a simulation box comprising 650 distinct bead-spring polymers. We apply *F*_act_ at each time step and study the phase behavior of polymers using multiple chain simulations. (**B**) Equilibrium snapshots from the simulations of two-phase systems for different strengths of the applied force *F*_act_ at the end of the simulation. (**C**) Radial density profile of the polymer beads from the center of the condensate. COM, center of mass. (**D**) Coexistence curves, derived from the radial density profiles, are used to delineate the two-phase regime bracketed by the coexisting dilute phase (left arm) and dense phase (right arm) at different values of *F*_act_. (**E**) Variation of ρ_sat_ as a function of *F*_act_ shows the log-linear relationship between these quantities. (**F**) Velocity of molecules in the dense phase plotted as a function of the magnitude of the active force *F*_act_. The units of velocity are σ/τ′, where τ′=200τ and τ is the natural unit of time. (**G**) Radial density profile of the polymer beads in the direction perpendicular to applied force (*z* = 0 plane) from the center of the condensate. (**H**) Radial density profile in the direction parallel to applied force from the center of the condensate (*x* = 0 plane and *y* = 0 plane). (**I**) Interfacial widths in the direction parallel and perpendicular to the applied force. The error bars denote the standard error of the mean over the course of the simulation trajectory.

We used Langevin dynamics simulations of model bead-spring polymers with beads interacting via a Lennard-Jones potential (Fig. 3A). The parameters of the model were chosen to ensure that the polymers can undergo spontaneous phase separation. To model the effects of motility, the simulations include an active force (*F*_act_) applied to the bead at one end of each of the polymers. The force is applied in the *z*-direction. The simulations probe how molecules that can undergo spontaneous phase separation respond to an applied force along a cardinal direction that approximates movement along the cytoskeleton. We performed multiple independent simulations at different magnitudes of the applied force (Fig. 3B and movie S4).

For a given value of *F*_act_, we compute the radial density profile (*26*), which helps quantify polymer densities within coexisting dilute (ρ_sat_) and dense phases (ρ_den_) (Fig. 3C). The width of the two-phase regime increases with increasing *F*_act_ (Fig. 3D). However, the dense phase concentration changes minimally, whereas the threshold concentration for phase separation, quantified as ρ_sat_, decreases by more than an order of magnitude for a fivefold increase in the magnitude of *F*_act_ (Fig. 3, D and E). The simulations show that the applied force, which enables active motions of polymers, enhances the driving force for phase separation.

We observed two distinct regimes for the impact of *F*_act_ on the overall phase behavior. In the weak-response regime, the dilute phase concentration changes minimally with increasing *F*_act_ (Fig. 3E). This is true for *F*_act_ ≤ *k*_B_*T*/σ, where *k*_B_*T* is the thermal energy and σ is the size of each bead on the polymer. In the strong response regime, *F*_act_ exceeds *k*_B_*T*/σ, and we observe a log-linear relationship between ρ_sat_ and *F*_act_ (Fig. 3E). Note that log(ρ_sat_) is proportional to the chemical potential of the polymer in the dilute phase. Therefore, the derivative of −*k*_B_*T* log(ρ_sat_) with respect to *F*_act_ is a measure of the conjugate variable, which is the net displacement of the molecules with respect to one another. Increasing *F*_act_ increases the net displacement, implying that the applied force drives motility (Fig. 3F), which facilitates associations of the polymers, and this in turn enhances the driving forces for phase separation.

The connection between enhanced motility and enhanced driving forces is evident in the lowering of ρ_sat_ (Fig. 3E) and the increased velocities of polymers in the dilute phase with increasing magnitude of *F*_act_ (Fig. 3F). The velocities of molecules in coexisting dilute and dense phases increase exponentially with increasing *F*_act_ (Fig. 3F and fig. S2). This is a manifestation of motility-enhanced phase separation, which is concordant with the observations for ARF19 molecules in the cytoplasms of plant cells.

### Applied forces lead to the breaking of spherical symmetry at the interface

For different values of *F*_act_, we computed radial density profiles in directions parallel and perpendicular to the interfaces between dense and dilute phases (Fig. 3, G and H). These profiles were used to compute the interfacial widths parallel and perpendicular to the interface (Fig. 3I). If the width of the interface is ∆, then the interfacial free energy density is proportional to (1/∆^2^) (*27*). In the strong-response regime, *F*_act_ > *k*_B_*T*/σ, the width of the interface parallel to the applied force increases with increasing *F*_act_. This implies that interfacial free energy density is lowered parallel to the applied force. Conversely, the width of the interface decreases in the direction perpendicular to the applied force, implying that the interfacial free energy density increases in this direction. Increasing the magnitude of the directional force *F*_act_ enhances the driving forces for phase separation and breaks spherical symmetry at interfaces (Fig. 3I), leading to dense phases that become increasingly aspherical (Fig. 3B and movie S4). This is because the molecules preferentially align parallel to the applied force (Fig. 3B and movie S4). It is noteworthy that asphericities of condensates have been used to extract viscoelastic moduli of cytoplasmic condensates (*28*). This rests on the assumption that condensates form and dissolve purely via passive forces. Our results suggest that asphericities of condensates might also point to the contributions from motility as modulators or enhancers of condensation.

### Dense phases concentrate active molecules in mixtures of active and passive species

We investigated the effect of introducing passive polymers in the system to test how motility-enabled phase separation is influenced by the presence of passive molecules. Only the active polymers experience the applied force (*F*_act_), whereas the passive polymers undergo Brownian motion. The relevant parameter is *f*_passive_, which is the fraction of polymers that are passive. For *f*_passive_ = 0, all the polymers in the simulation experience the applied force *F*_act_. For *f*_passive_ = 0.05, 5% of the molecules undergo purely Brownian motion.

**Fig. 4.**
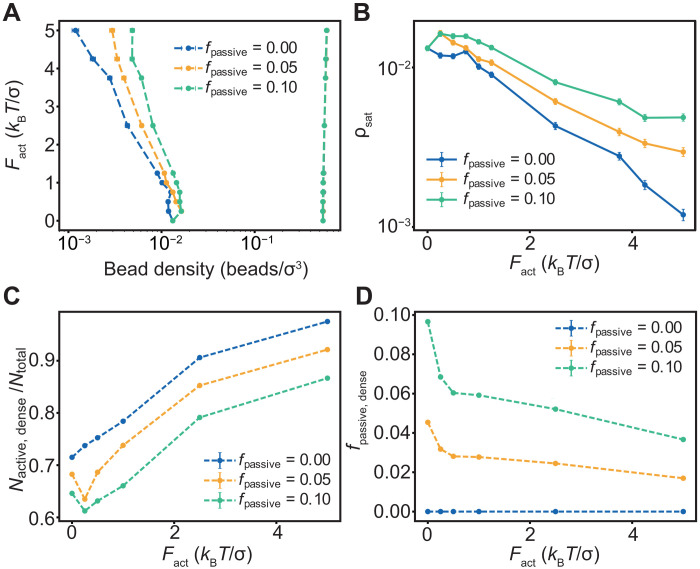
Passive molecules are left behind in the dilute phase as *F*_act_ increases in magnitude. (**A**) Phase boundaries for an active-passive mixture as a function of active force for different fractions of passive chains *f*_passive_. (**B**) ρ_sat_ as a function of the active force *F*_act_ for different fractions of the passive polymers *f*_passive_. (**C**) Number of active chains in the dense phase normalized by total chains in the system as a function of the active force (*F*_act_) for different fractions of the passive polymers *f*_passive_. (**D**) Fraction of the passive chains in the dense phase as a function of the active force (*F*_act_) for different fractions of the passive polymers *f*_passive_. The error bars are the standard error of the mean over the course of the simulation trajectory.

For a given value of *F*_act_, the width of the two-phase regime decreases as *f*_passive_ increases (Fig. 4A). Increasing *f*_passive_ competes with and diminishes the motility-driven enhancement of the driving force for phase separation. The net displacement of molecules toward one another decreases as *f*_passive_ increases. This is clear from the decrease in the slope of the log-linear regime of the plot of ρ_sat_ against *F*_act_. In a mixture of active and passive molecules, there is selective partitioning of active molecules into dense phases, and this depends on the magnitude *F*_act_. The fraction of active molecules that partition into the dense phase increases with increasing *F*_act_ (Fig. 4C), whereas the fraction of passive molecules that partition into the dense phase decreases with increasing *F*_act_. Overall, the dense phase accumulates molecules that are drawn to one another by enhanced motility. Therefore, the selective application of force breaks symmetry by distinguishing molecules from one another. This in turn leads to preferential accumulation of active molecules in dense phases and passive molecules in dilute phases.

## DISCUSSION

Actomyosin network–directed motion of molecules is a key feature of cells, which are active, out-of-equilibrium systems (*29*–*31*). Motility-induced phase separation has been studied in the context of Brownian particles moving at high speeds under the influence of applied forces (*32*). Two types of phenomena are of interest. They are the clustering of noninteracting, self-propelled particles (*32*) and the clustering of motile, albeit sticky, particles (*33*). The ARF system belongs to the latter category. Furthermore, the effective temperature in the vicinity of motile particles is likely to be higher than the bath temperature of the cell (*34*). This likely has consequences on the thermoresponsive phase behaviors of other condensate-forming systems (*35*) that are in the spatial proximity of motile condensates.

Our computations focused on how applied forces alter the underlying phase behaviors of sticky, linear polymers. We find two regimes for the interplay between the driving forces for phase separation, measured in terms of the change in saturation concentrations, and the magnitude of the applied force. In the strong-response regime, we observe a log-linear relationship between ρ_sat_ and *F*_act_. Increases in the applied force increase the displacements of polymers, and this augments cohesive interactions, thereby lowering ρ_sat_. Our observations appear to be an example of motility-aided phase separation (*36*, *37*). The simulations also show that as the applied force increases, there is a clear segregation between active and passive molecules, with the former being enriched in dense phases and the latter in dilute phases.

ARF condensates mature over time and older cells contain condensates that show less internal mobility, as measured by fluorescence recovery after photobleaching, when compared to younger cells (*5*). The material properties of ARF19 condensates can be influenced by changes to the amino acid composition of disordered regions within the ARF19 protein (*38*). The current thinking is that nonequilibrium considerations in live cells will have an impact on the material properties, aging, and compositions of condensates (*39*, *40*). It remains to be seen whether condensate motility varies across the root system and whether this, in conjunction with passive considerations, influences ARF condensation as a function of space and time. The implication of motility-enabled condensation on the work done by condensates (*41*–*43*) or the role of condensates as mechanosensory elements (*44*) is likely to be an important aspect of cell physiology.

The observed rapid movement of ARF condensates is driven by myosin XI activity. Because the high speed of these plant myosins is driven by fast adenosine 5′-triphosphate hydrolysis rates (*18*), these condensate movement rates are unlikely to be achievable in other systems; however, the underlying effect of movement-generated force should be the same. Furthermore, although plant actin filaments are randomly arranged during interphase (*45*), the dense cortical network of animal systems has the potential to have a concentrating effect on condensates moved along actin filaments in these other systems.

The unique characteristic of the ARF system in displaying elevated dilute concentrations in the nucleus and an absence of persistent condensates and reduced dilute concentrations in the cytoplasm with mobile condensates has allowed us to unmask a driver of condensation; however, this driver is unlikely to be limited to the ARFs. Previous studies showed that processing bodies (P-bodies), which are RNA storage and processing condensates, are also motile in the plant cell cytoplasm. P-bodies move along the actin cytoskeleton under the influence of direct interactions between decapping protein 1 (DCP1) and myosin XI (*46*). Similar to our findings with ARF condensates, P-body size was dependent on movement (*46*), suggesting that active transport also enables protein condensation in this system. A variety of plant viruses is known to form cytoplasmic condensates in plant cells. These viral condensates move along microfilaments, aided by different myosins (*47*, *48*). For example, mobile condensates are formed by the P protein of the *Barley yellow stripe mosaic virus* (BYSMV) in plant cells (*49*). These condensates are trafficked along the actin cytoskeleton, and this movement helps with the recruitment of viral N and L proteins. Although these previous studies did not connect condensation and motility as we have done, the principles we describe here likely also underlie recruitment to these motile biomolecular condensates. Thus, our combination of in-cell investigations and modeling provides a physical basis for the observed phenomenology. Our results suggest that inhibiting motility by directly targeting the interactions between viral proteins and myosins might provide a route disrupting viral assembly replication.

At the outset, we discussed four possibilities for the observation of condensates in the cytoplasm versus nucleus despite the dilute phase concentration being higher in the nucleus. Of the four options, we investigated motility-influenced phase separation in the cytoplasm because we observed directed motions of condensates. To investigate the possibility that condensation within the nucleus might be inhibited by active processes, it will be essential to assess whether transcriptional or other nuclear activities drive negative feedback responses that suppress condensation. Ligand effects also remain a formal possibility. Knowledge of the interactors of ARFs, which identifies putative ligands, coupled with systematic knockdowns of the ligands will be helpful in testing whether ligand binding enables or suppresses condensation in different regions. The other likely scenario, in addition to motility-influenced condensation in the cytoplasm, remains the possibility that the intracellular solution conditions are sufficiently different between the nucleus and the cytoplasm in plant cells. The overall strengths of intermolecular interactions and whether they are attractive or repulsive will depend, at least to the first order, on the magnitude and sign of the second virial coefficient (*B*_2_). It is likely that *B*_2_ is either less negative or even positive in the nucleus when compared to the cytoplasm. Adjudication of this matter will require measurements of *B*_2_ in nuclear versus cytoplasmic extracts. If *B*_2_ is less negative in the nucleus than the cytoplasm, then differences in solution conditions between the nucleus and the cytoplasm can become additionally relevant because they can generate passive ion and metabolite gradients across the nuclear membrane. The presence of ion gradients can accelerate the transport of macromolecules in confined spaces such as microfluidic channels (*50*). The result is local concentration inhomogeneities, whereby diffusiophoresis drives locally supersaturated regions to promote the formation of condensates in location-specific ways. Encountering ion or metabolite gradients might enable intranuclear condensation of ARFs via diffusiophoresis. It might also drive the ejection of condensates from the nucleus to the cytoplasm. Whether condensates translocate from the nucleus to the cytoplasm, and how this happens, remains to be investigated.

Principles that are similar to what we have uncovered might apply to other condensates defined by electrochemical gradients (*51*) including nucleoli (*8*). Here, local pH gradients might be drivers of the proposed advective transport of ribosomal RNA molecules (*52*), thus contributing to activity-dependent maintenance of the multiphasic structures of nucleoli (*53*). Motor- or field-driven transport of macromolecules might be a common way for cells to drive condensate formation and condensate transport. Our findings, together with previous work and theories of motility-induced phase separation (*37*), might be directly relevant for a range of phase-separating systems in cells, and this topic merits detailed investigations given their potential functional relevance in plant, fungal, and mammalian cells.

## MATERIALS AND METHODS

### Growth conditions and phenotypic assays

*A. thaliana* ecotype Colombia (Col-0) was the genetic background for all reporters used in this study. Surface-sterilized seeds were stratified overnight at 4°C and plated on plant nutrient medium solidified with 0.6% (w/v) agar and supplemented with 0.5% (w/v) sucrose. Seedlings were grown horizontally at 22°C with continuous illumination.

To examine ARF condensate movement, multiple reporters were used, including pUBQ10:YFP-ARF19 (*5*), pARF19:ARF19-mVenus, and pARF7:ARF7-mVenus. pUBQ10:YFP-ARF19 was crossed to myosin triple mutant (*3KO*; *myosin xi-1 xi-2 xi-k*). This triple mutant was previously generated using SALK insertion lines in XI-K (SALK_067972; At5g20490), XI-1 (SALK_019031; At1g17580), and XI-2 (SALK_055785; At5g43900) (*54*, *55*).

To examine the effects of disrupting the cytoskeleton, we incubated 4-day-old seedlings in water supplemented with the indicated concentrations of oryzalin, LatB, or an equivalent volume of the carrier dimethylsulfoxide (DMSO) for 2 hours before imaging. Seedlings were mounted on slides in water supplemented with the indicated treatment.

To examine ARF19 condensate movement in tobacco, *Agrobacterium* strain GV3101 was transformed with pUBQ10:YFP-ARF19 and LifeACT-mCherry (*56*) binary vectors. *Agrobacterium* cells were collected by centrifugation, then washed, and resuspended to an optical density at 600 nm of ~1.0 in infiltration medium [10 mM MES (pH 5.6), 10 μM MgCl_2_, and 1 μM acetosyringone]. Leaves of 3-week-old *Nicotiana tabacum* were infiltrated with *Agrobacterium* carrying the expression vectors and imaged 36 hours after postinfiltration.

### Microscopy

Four-day-old seedlings carrying the *pARF7:mVenus-ARF7* (*5*), *pARF7:mVenus-ARF19* (*5*), or *pUBQ10:YFP-ARF19* (*5*) reporters were mounted in water on glass slides under a coverslip and imaged using a Leica DMi8 confocal microscope through a 40× water immersion lens. For uniform analysis, all imaging used for quantification of condensate speed was from immature root hair cells to control the developmental age and ARF condensate aging. Images of wild type and *myosin xi-k xi-1* (*myo2KO*) or *myo3KO* carrying *pUBQ10:YFP-ARF19* were taken of the first visible condensates to within the differentiation zone of the root. Condensate area was measured using the calculate area function in Fiji (*57*).

TrackMate (*58*) was used to analyze condensate movement and velocity. Spots were detected using a DoG detector that permits subpixel localization. Spots were then filtered on the basis of quality and tracked using the Lap Tracker. Linking conditions and gap-closing conditions were toggled to eliminate biological noise but never permitting above a maximum distance of 15.0 pixels. Tracks were then manually pruned to eliminate misannotated spots. The *x*- and *y*-coordinates between frames were then used to determine the change position and angle between spots to determine the instantaneous velocity per spot.

### Local MSD analysis

We used the local MSD analysis to distinguish active versus passive condensate motions. Using the method described by Arcizet *et al.* (*17*), we calculated local MSDs with a time window of 12 time steps (each with a size of 0.15 s) to delineate the active versus passive motions.

### Computer simulations of active polymers

We used the LAMMPS package (*59*) for Langevin dynamics simulations of polymers to study the role of activity on phase separation of polymers. We modeled polymers as flexible macromolecules with bead-spring architecture. Adjacent beads within polymers were connected by the finitely nonlinear elastic bond potential (*60*): $U_{\text{FENE}}\left(r\right)=-\frac{1}{2}KR_{0}^{2}\text{ln}\left[1-{\left(\frac{r}{R_{0}}\right)}^{2}\right]$ and *K* = $100\;\frac{\epsilon }{\sigma^{2}}\;$. All nonbonded monomers within each chain and between chains interacted via a Lennard-Jones potential $U\left(r\right)=4\epsilon \left[{\left(\frac{\sigma }{r}\right)}^{12}-{\left(\frac{\sigma }{r}\right)}^{6}\right]\;$ with ϵ = 0.40$k_{B}T$. To mimic activity, a force *F*_act_ was applied to the head bead of a polymer in the *z*-direction. Hence, the conservative force because of the Lennard-Jones potential and finitely nonlinear elastic potential was supplemented by the active force *F*_act_ for the active beads. For the simulations of mixtures of active and passive polymers, the passive polymers lacked the active head bead and, instead, the head bead was purely passive.

The Langevin equation was integrated forward in time using the velocity-Verlet algorithm (*61*). The time step for integration was chosen to be 0.01τ, where τ is the natural unit of time. The damping parameter was chosen to be 0.50τ. We used a total of 650 chains with 25 beads each and a simulation box size of 64 by 64 by 100, spanning *x* = [−32,32], *y* = [−32,32], and *z* = [−50,50]. The chains were initialized randomly in the cubic box, and a biasing harmonic potential was applied, for 2 × 10^5^ time steps, to each bead to obtain a single dense phase. We then turned off the biasing potential and performed Langevin dynamics simulations in the presence of an applied force *F*_act_ that acts along the *z*-direction. Each simulation was performed for a total of 5 × 10^7^ time steps, and information for the last 2.5 × 10^7^ time steps was used for analysis. The simulation snapshots were saved every $2\times 10^{4}$ time steps. We used OVITO (*62*) to visualize the simulation trajectories.

### Calculation of radial density profiles and coexisting phase concentrations

We calculated the radial density profile of the entire system from the center of mass of the identified largest cluster and obtained sigmoidal radial density profiles with the radial density at small distance *r* denoting the dense phase densities and dilute phase densities at large distance *r*. We calculated the radial density profile for all atoms in the *z* = 0 plane to obtain radial density in the direction parallel to the active force. For obtaining the radial density perpendicular to the active force, we calculated the radial densities for all atoms in the *x* = 0 plane and *y* = 0 plane.

We used the derivatives of the radial density profile to obtain the interfacial widths. This derivative should be zero in the dense and dilute phases. By using this property, we calculated the interfacial widths by calculating the size of the region where the radial density profile is nonzero. We obtained the maximum and minimum radial distances where the derivative is monotonically increasing and monotonically decreasing to characterize the interfacial region. Using bootstrapping with 10^3^ trials, wherein one random data point was skipped in each trial, we obtained mean and SDs of interfacial widths.

### Calculation of area for each condensate at each snapshot

For a given snapshot, we use the TrackMate-derived *x*- and *y*-positions of each condensate as a starting point. We make a window around the pixel corresponding to the condensate localized using TrackMate. TrackMate data that do not correspond to a frame at a known time point given the temporal resolution are excluded from the automated image analysis performed in MATLAB (r2023b). In all cases, we chose the window for each subimage to be ±5 pixels about the central pixel. This ensures that the putative condensate falls entirely within each subimage given the magnification. Condensates are excluded from subsequent analysis only if a sufficiently large window cannot be constructed for a condensate at the edge of the frame. We then binarize each subimage via global thresholding at 33% of the intensity of the pixel of maximum intensity in the subimage. We associate the condensate with the connected component that is nearest to the central pixel after thresholding. We then fit an ellipse using the function “regionprops” from MATLAB’s Image Processing Toolbox, which gives us both the major and minor axes of the ellipse. The area of each condensate is determined by *A* = π(length of major axis/2)(length of minor axis/2). We convert the area in pixels to real units by multiplying this value by the pixel resolution for the given dataset.
