# Supplementary material for: Active transport enables protein condensation in cells
Source: Sci Adv. 2025 May 23;11(21):eadv7875. doi: 10.1126/sciadv.adv7875 (PMC12101484; doi:10.1126/sciadv.adv7875)
Supplement: Supplementary file 1 — Figs. S1 to S4 Legends for movies S1 to S4 [file sciadv.adv7875_sm.pdf]

Science Advances
AAAS

Supplementary Materials for

Active transport enables protein condensation in cells

Gaurav Chauhan *et al.*

Corresponding author: Rohit V. Pappu, pappu@wustl.edu; Lucia C. Strader, lucia.strader@duke.edu

Sci. Adv. **11**, eadv7875 (2025)
DOI: 10.1126/sciadv.adv7875

The PDF file includes:

Figs. S1 to S4
Legends for movies S1 to S4

Other Supplementary Material for this manuscript includes the following:

Movies S1 to S4

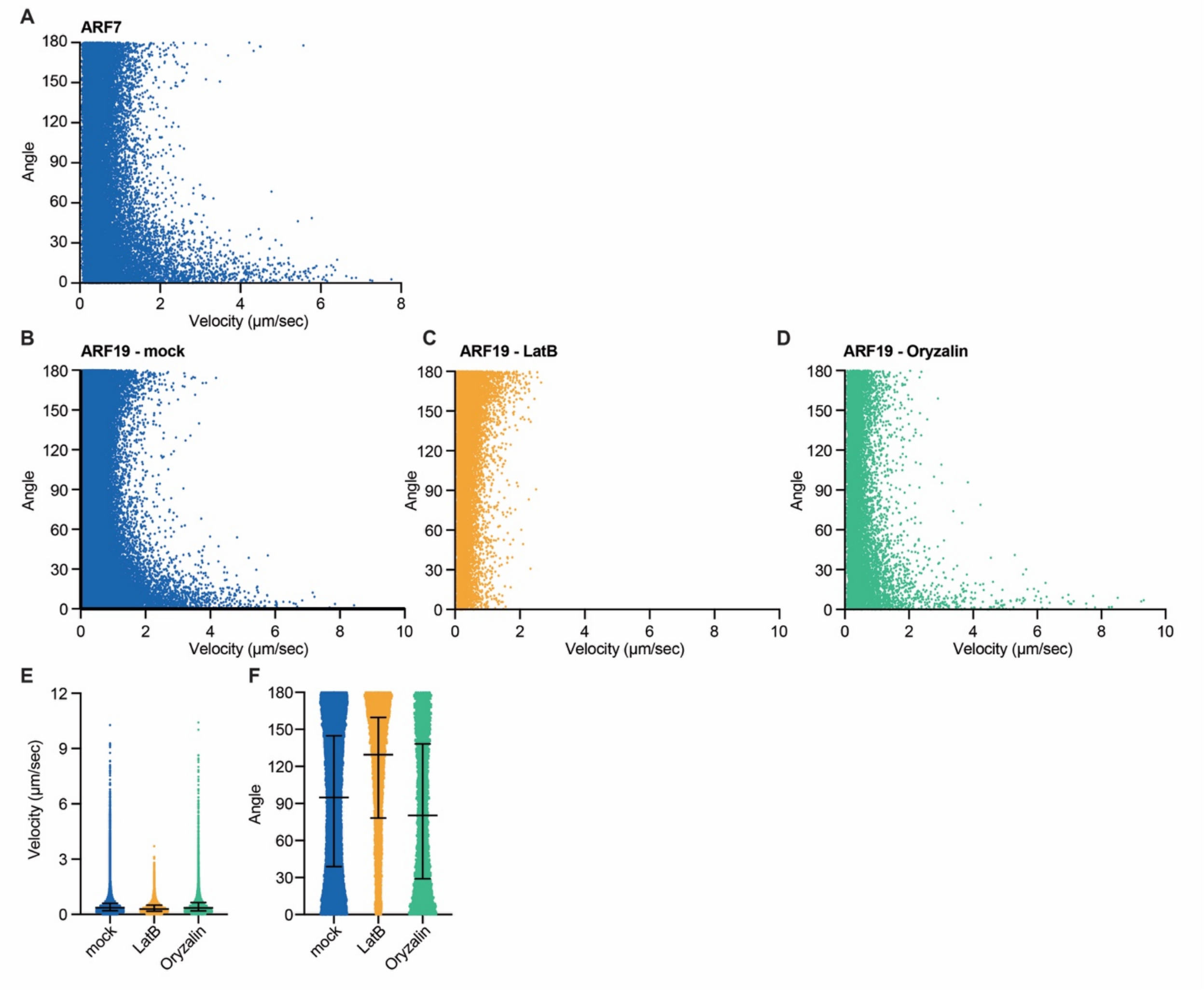

Fig. S1. LatrunculinB disrupts ARF condensate movement

(**A**) Angle and speed scattergrams of ARF7-mVenus condensates from seedlings expressing *pARF7:ARF7-mVenus*. Each angle and velocity spot was determined using an average of 3 frames collected over 1.31s. Analysis from 11 different individuals and 7937 total spots. (**B**, **C**, **D**) Angle and speed scattergrams of ARF19-mVenus condensates from seedlings carrying *pARF19:ARF19-mVenus* treated with a 2-hour (B) mock (DMSO) (C) 10 µM LatB, or (D) 40 µM Oryzalin treatment. Each angle and velocity spot was determined using an average of 3 frames collected over 1.31s Analysis were from ≥5 cells from ≥ 3 individuals for ≥10557 total spots. (**E**) Distribution of condensate velocity within each treatment: mock, 10 µM LatB, or 40 µM Oryzalin. (**F**) Distribution of condensate angles within each treatment: mock, 10 µM LatB, or 40 µM Oryzalin.

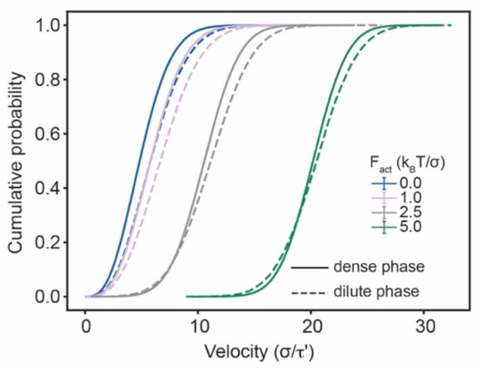

Fig. S2. Cumulative probability distribution (CDF) for the velocity of chains in the dense and dilute phase, at different magnitudes of the active force

In general, the probability that molecules move at higher speeds increases with increasing value of the applied force. For low force, the CDF is right shifted in the dilute phase, implying that the molecules in the dilute phase lag the molecules in the dense phase. This changes when F_{act} increases substantially beyond thermal energy.

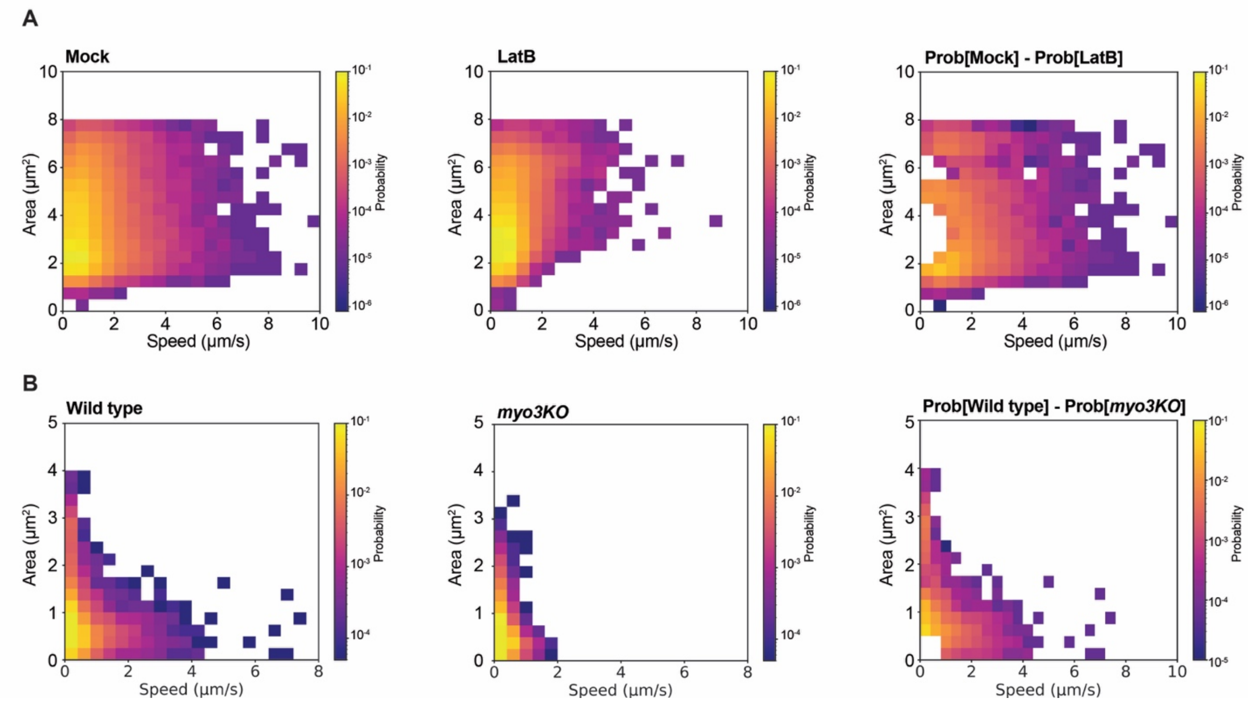

Fig. S3. Altered movement affects ARF condensate joint probability densities

(**A**) Joint probability densities for areas and speeds of the tracked pARF19:ARF19-mVenus condensates from seedlings incubated for two hours with a mock (DMSO) or 10 µM LatB treatment. Data collected using Trackmate (*6*) are shown for five distinct condensates captured from immature trichoblasts over the course of five minutes at a frame rate of 12 frames/second. (**B**) Joint probability densities for area and speeds of YFP-ARF19 condensates in young root epidermal cells of wild type and *myosin xi-k xi-1 xi-2* (*myo3KO*) seedlings carrying *pUBQ10:YFP-ARF19*.

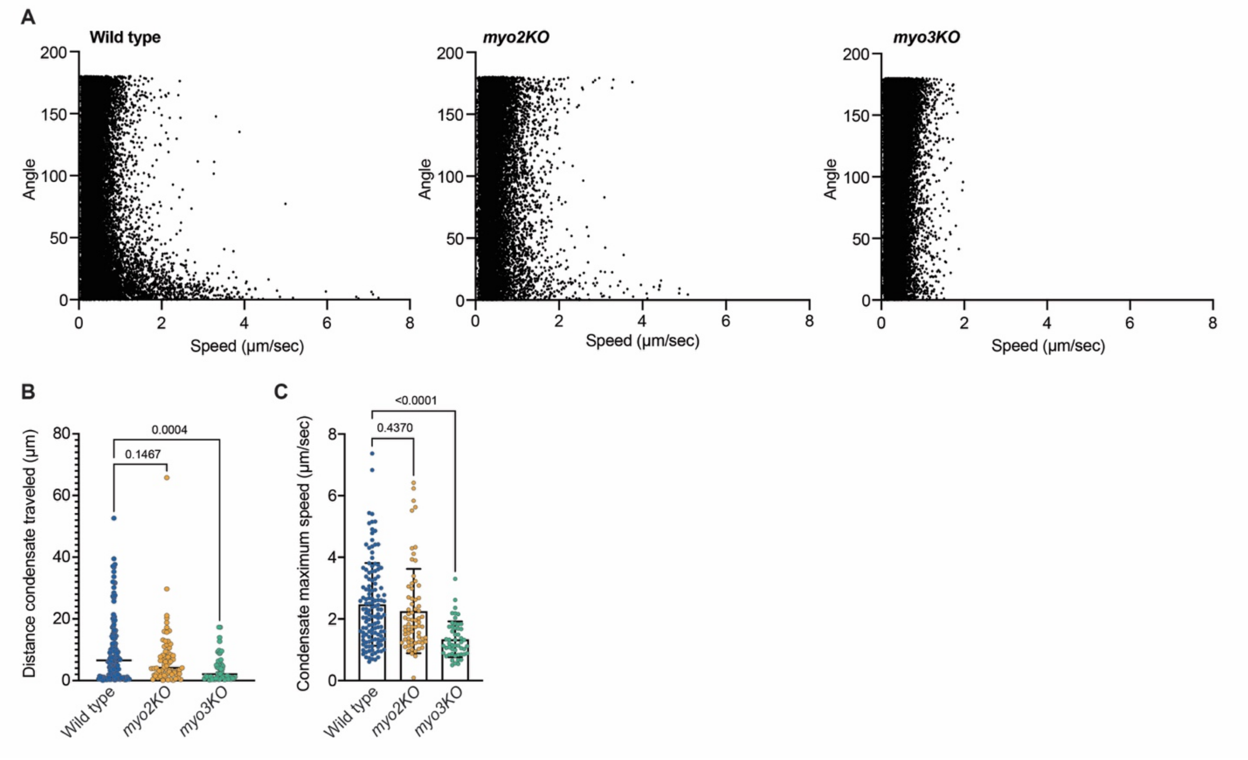

Fig. S4. Myosin 3 is required for ARF condensate movement

(**A**) Speed versus angle distribution YFP-ARF19 condensates in young root epidermal cells of wild type, *myosin xi-k xi-1* (*myo2KO*), and *myosin xi-k xi-1 xi-2* (*myo3KO*) seedlings carrying *pUBQ10:YFP-ARF19*. (**B**) Observed distance traveled by an individual condensate during the observation period. Images were acquired every 0.38s for 5 minutes. Condensates were followed using Trackmate. (**C**) Observed maximum speed of a condensate as it traveled within a cell. Images were acquired every 0.38s for 5 minutes. Condensates were followed using Trackmate. N≥52 condensates analyzed per genotype. Statistics analyzed using an ordinary one-way ANOVA.

Movie S1. ARF condensate movement

A series of timelapse confocal images of YFP-ARF19 signal in root epidermal cells of Col-0 carrying *UBQ10:YFP-ARF19*. Images were taken of the transition zone in which condensates first start to appear. In addition, a second series of timelapse confocal images of ARF19-mVenus signal in root epidermal cells of *arf19-1* carrying *ARF19:ARF19-mVenus*.

Movie S2. ARF condensates move along actin filaments

A series of timelapse confocal images of ARF7 condensates moving along actin filaments in an Arabidopsis root hair and ARF19 condensates moving along actin filaments in tobacco epidermal cells.

Movie S3. Effects of myosin knockout on ARF condensate movement

Timelapse confocal imaging of ARF19-mVenus signal in root epidermal cells of arf19-1 carrying ARF19:ARF19-mVenus treated for the indicated time with Mock (DMSO) or 10 µM LatB. Timelapse confocal imaging of YFP-ARF19 signal in young root epidermal cells of wild type and *myosin xi-k xi-1 xi-2* (*myo3KO*) seedlings carrying *pUBQ10:YFP-ARF19*.

Movie S4. Sample LAMMPS simulation trajectories

Sample LAMMPS simulation trajectories of the two-phase polymeric system with the head bead of the polymers subjected to the active force of the magnitude: a) F_{act}= 0, b) F_{act}= 1 k_BT/σ, c) F_{act} = 2.5 k_BT/σ , d) F_{act}=5 k_BT/σ. Each trajectory consists of 60 frames, each separated by 20000 timesteps.
